# Artificial Intelligence in Point-of-Care Biosensing: Challenges and Opportunities

**DOI:** 10.3390/diagnostics14111100

**Published:** 2024-05-25

**Authors:** Connor D. Flynn, Dingran Chang

**Affiliations:** 1 Department of Chemistry, Weinberg College of Arts & Sciences, Northwestern University, Evanston, IL 60208, USA; 2 Department of Biomedical Engineering, McCormick School of Engineering, Northwestern University, Evanston, IL 60208, USA

**Keywords:** biosensor, point-of-care, artificial intelligence, machine learning

## Abstract

The integration of artificial intelligence (AI) into point-of-care (POC) biosensing has the potential to revolutionize diagnostic methodologies by offering rapid, accurate, and accessible health assessment directly at the patient level. This review paper explores the transformative impact of AI technologies on POC biosensing, emphasizing recent computational advancements, ongoing challenges, and future prospects in the field. We provide an overview of core biosensing technologies and their use at the POC, highlighting ongoing issues and challenges that may be solved with AI. We follow with an overview of AI methodologies that can be applied to biosensing, including machine learning algorithms, neural networks, and data processing frameworks that facilitate real-time analytical decision-making. We explore the applications of AI at each stage of the biosensor development process, highlighting the diverse opportunities beyond simple data analysis procedures. We include a thorough analysis of outstanding challenges in the field of AI-assisted biosensing, focusing on the technical and ethical challenges regarding the widespread adoption of these technologies, such as data security, algorithmic bias, and regulatory compliance. Through this review, we aim to emphasize the role of AI in advancing POC biosensing and inform researchers, clinicians, and policymakers about the potential of these technologies in reshaping global healthcare landscapes.

## 1. Introduction

Point-of-care (POC) bioanalysis continues to revolutionize patient care by bringing sophisticated diagnostic capabilities directly to the patient whether at the bedside, in the field, or at home [1]. These systems are enabled by a broad range of sensing strategies that provide sensitive and accurate information rapidly without the need for centralized testing. Biosensing has become increasingly useful as a means to obtain POC measurements due to the high selectivity and sensitivity that these systems provide [2]. Through the incorporation of naturally occurring biorecognition elements as receptors, it is possible to measure glucose levels [3], diagnose viral infections [4], and even identify cardiac events [5] in only a few minutes. However, the development of new biosensing tools is no simple task. Each step of the sensor development process is plagued with difficulties that require careful consideration and often complex solutions. Artificial intelligence (AI) has immense potential to assist with these challenges through the application of data-driven computational approaches that provide unparalleled predictive power. With the incorporation of AI into biosensing, a new generation of POC technologies is on the horizon. 

The term *artificial intelligence* was coined during a summer research project at Dartmouth in 1956 [6]. It refers to the development of computational approaches that can approach or surpass human intelligence. While many areas of AI are still theoretical, the continued development of areas like machine learning has begun to bring these concepts into reality. With the recent emergence of generative AI applications (e.g., OpenAI’s ChatGPT [7]), the power of AI is on full display and interest in the field is at an all-time high. When it comes to science, the power of AI lies in its ability to extract patterns and trends from large datasets—a feat not easily accomplished by the human brain. Using this information, it is possible to optimize protocols, interpret results, and predict outcomes with better accuracy than ever before.

This review aims to highlight the many avenues through which AI stands to benefit the biosensing community at every stage of the biosensor development process. Given that many previous reviews on this subject are either overly complex on the computational side or oversimplified when describing AI biosensor integration, we seek to strike a middle ground that is beneficial to those from computational, diagnostic, and biosensing backgrounds. We provide a thorough breakdown of biosensor development and its application in point-of-care bioanalysis, followed by a discussion of the AI approaches that are currently being implemented in the field. We finish the review with an exhaustive list of biosensing-related challenges that AI can help with in coming years, as well as an outlook for the foreseeable future.

## 2. What Is a Biosensor?

**Figure 1:**
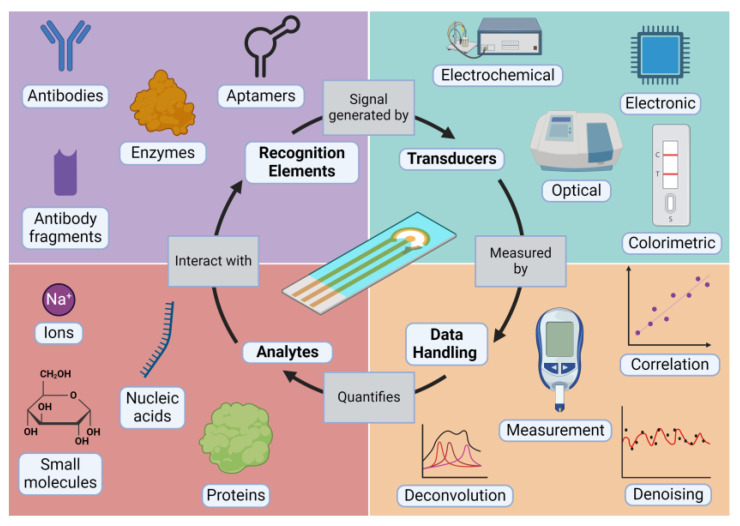
The biosensor development process.

Biosensors are bioanalytical systems named for their use of biorecognition elements to interact with and identify target analytes [2]. A biosensor is typically comprised of four main components: the *analyte*, which is the molecule of interest that is detected using the biosensor; the *recognition element*, which is the molecule or structure that interacts with the analyte; the *transducer*, which is the instrument or method that converts the interaction between the analyte and recognition element into a readable signal; and the *data handling* procedures, which allow for analysis and interpretation of the generated data. The components of a biosensor are described below and presented in Figure 1.

### 2.1. Analytes

An analyte is any entity that may be targeted by a biosensor. Analytes may comprise ions [8], small molecules [9], nucleic acids [10], proteins [11], viruses [12], bacteria [13], or even whole human cells [14]. These entities are typically biomarkers of human health or disease but may also be exogenously introduced drugs or other clinically relevant molecules. Outside of the human body, analytes may be relevant to monitoring water/soil quality, assessing food contamination, or discovering new drugs [15].

Analyte selection is essential to the development of practical biosensors. While there is no real limitation on what can be considered an analyte, there are several factors that should be considered when determining a target. Firstly, an analyte should be relevant to the application for which the biosensor is being developed (i.e., select an analyte with an established link to the disease of interest). Secondly, the analyte should be present and at a sufficient concentration in the biological fluid to be assessed (i.e., select an analyte known to be present in saliva for a saliva-based biosensor). Thirdly, variations in the concentration of the analyte should reflect some physiologically relevant parameter (e.g., an increase in the analyte indicates an exacerbated disease state). Finally, an analyte should have a known, selective recognition element to enable its targeting. 

### 2.2. Recognition Elements

The incorporation of biologically derived recognition (biorecognition) elements into bioanalytical systems provides unrivaled analyte specificity. These receptors are often the product of millions of years of evolution and their interactions form the basis for life itself. Biorecognition elements may be either catalysis-based or affinity-based, depending on whether they react with or bind to the analyte of interest, respectively. Synthetic recognition elements composed of manmade materials may also be employed in biosensors. The most common recognition elements can be divided into three main categories: protein-based, nucleic acid-based, and artificial.

Protein-based recognition elements include all entities formed from amino acids, the most common of which is the enzyme. Enzymes are proteins capable of catalyzing reactions in the presence of specific substrates in a simple and rapid manner. The massive success of commercial glucose sensing can largely be attributed to the existence of redox enzymes like glucose dehydrogenase and glucose oxidase, which react with glucose with high selectivity [16]. However, there is a lack of similarly selective enzymes for most other analytes, which complicates enzyme-based sensing.

Antibodies are another protein-based recognition element that serve as the gold standard in affinity-based recognition [17]. Antibodies are produced in the body by B cells in response to antigen-induced cues, conferring them with a high degree of specificity for their target. Alternatively, sections of antibodies or antibody analogs from other animals may be engineered together into high-affinity binders known collectively as antibody fragments [2]. These fragments (e.g., short-chain variable fragments (scFvs) [18] and nanobodies [19]) are often much smaller than antibodies and can be modified to suit various applications (e.g., pH resistance, chemical modification).

Nucleic acid-based recognition elements include all entities formed from nucleic acids, including DNA, RNA, or other synthetics (e.g., PNA). At the simplest level, nucleic acids can serve as receptors for their complementary sequence when targeting nucleic acid analytes (e.g., microRNAs [10]). For other targets, nucleic acids that preferentially bind to specific targets—known as aptamers—may be employed [20]. Aptamers are selected through the systematic evolution of ligands by exponential enrichment (SELEX) [21,22], whereby large pools of nucleic acid sequences are subjected to evolutionary pressure via the elimination of non-binding sequences until only those that bind the analyte strongly remain. Alternatively, nucleic acids that exhibit catalytic activity (e.g., DNAzymes [23], ribozymes [24]) may also be used to react with specific analytes.

Artificial recognition elements are unique as they are formed from synthetic materials instead of natural biomolecules. Because the term biosensor implies a recognition element that is biological in origin, the broader term biomolecular sensor is becoming more popular as a way to describe all sensors used to detect biologically relevant molecules [2]. Artificial recognition elements include nanomaterial-derived catalysts (e.g., nanozymes [25]) and molecularly imprinted polymers (MIPs [26]).

### 2.3. Transducers

Transduction is key to biosensing systems as it provides a means to convert molecular interactions into signals that are more easily processed and analyzed. The most common transduction methods include electrochemical [11,13], electronic [27], colorimetric [28,29], optical [30], acoustic [31], thermal [32], and piezoelectric [33] methods.

Electrochemical transduction methods rely on the transfer of electrons, either between an electrode and the analyte or an electrode and an external redox reporter (i.e., a molecule that can be reversibly oxidized and reduced). Electrochemical techniques such as chronoamperometry [11,34], square wave voltammetry [35,36], and electrochemical impedance spectroscopy [13,37] can provide information on the electrical parameters (i.e., current, potential, impedance) that correlate with analyte concentration. Electronic transduction methods also operate on the basis of electrical parameter modulation; however, these approaches do not involve direct electron transfer (e.g., field-effect transistors [38]).

Colorimetric transduction involves the generation or alteration of color, typically via dyes or other colored reagents. This color change may be quantified with an instrument (e.g., spectrophotometer) or simply assessed by eye (e.g., pregnancy test strip [39]). Optical transduction methods are more complex and involve various light phenomena (e.g., fluorescence, evanescent waves) that contain information that correlates with analyte concentration [30].

Acoustic, thermal, and piezoelectric transduction involves the conversion of biomolecular interactions into sound, heat, or mechanical stress, respectively. While these methods tend to be less popular than others for POC biosensing, they form the foundation for many important biochemical characterization techniques, like quartz crystal microbalance and isothermal titration calorimetry [31,32,33]. 

### 2.4. Data Handling

Data handling is often overlooked in biosensor development and includes the acquisition, analysis, and interpretation of data. Basic instrument software is typically used to collect data in laboratory settings, which are later analyzed by hand or by using rudimentary scripts. While such practices are sufficient for laboratory-localized experimentation, the translation of biosensors from the lab to the real world requires the careful integration of data handling and interpretation procedures into self-sufficient platforms capable of independent analysis. 

At the simplest level, many colorimetric biosensors can be analyzed by eye without the need for further instrumentation. However, most other transduction methods require some form of device. These devices must be capable of measuring the data provided by the transduction mechanism, modifying the obtained data (e.g., deconvolution, amplification), and interpreting the data (i.e., converting signal into analyte concentration). Commercial biosensors, like portable glucose meters, have carefully engineered platforms that can seamlessly analyze samples; however, these platforms are often proprietary and inaccessible to the wider scientific community, leaving researchers to construct their own or rely on industrial engineers to complete this task after a technology has been acquired. 

## 3. Sensing at the Point-of-Care

Biosensors have become increasingly important in POC settings due to their ability to deliver immediate results at the site of patient care. Traditional diagnostic processes often require patients to visit healthcare facilities, collect samples, and send these samples to centralized laboratories for analysis. This can result in prolonged waiting periods—days or even weeks—for results, which significantly impedes timely decision-making and immediate patient care. Moreover, laboratory facilities, typically located in urban centers, are not readily accessible to those in remote or underdeveloped areas, posing challenges for healthcare access. In recent years, POC sensing technologies, including lab-on-a-chip platforms [40], microfluidic devices [41], paper-based sensors [42], and wearable or implantable sensors [43], have seen significant advancement. These technologies not only provide rapid results, enhancing the efficiency of medical interventions, but are also more user-friendly and cost-effective, and are able to be used at home. Such shifts in sensor development are crucial for enhancing health surveillance, enabling routine health monitoring, and improving global health outcomes. In this section, we will discuss the development and deployment of biosensors in POC applications, whether at the bedside, at home, or in the field.

### 3.1. Sensing at the Bedside

POC sensors are now routinely used in bedside clinical settings for rapid diagnostic screening and assessment. POC cardiac biosensors can be used to monitor biomarkers such as troponin T, D-dimer, and pro-B-type natriuretic peptide (proBNP) to pre-emptively identify cardiac events [44,45]. CRP biosensors can quickly measure C-reactive protein levels to assess inflammation or infection [46]. Coagulation sensors provide information on blood clotting times for patients on anticoagulant therapy [47]. Lactate biosensors measure lactate levels in patients with conditions like sepsis or shock [48]. Additionally, gas biosensors (e.g., for exhaled nitric oxide) can be employed to manage respiratory diseases such as asthma by assessing airway inflammation [49]. Biosensors are also used in the rapid identification of infectious pathogens to control the spread of infections and improve infection management. POC tests are available for various pathogens, including HIV [50], SARS-CoV-2 [51], and tuberculosis [52], and a significant research effort is devoted to expanding these tools [53,54,55]. The integration of these biosensors into clinical practice has significantly enhanced medical intervention efficiency and the future development of new biosensing technologies is expected to pave the way for a rapid medical turnaround.

### 3.2. Sensing at Home

**Figure 2:**
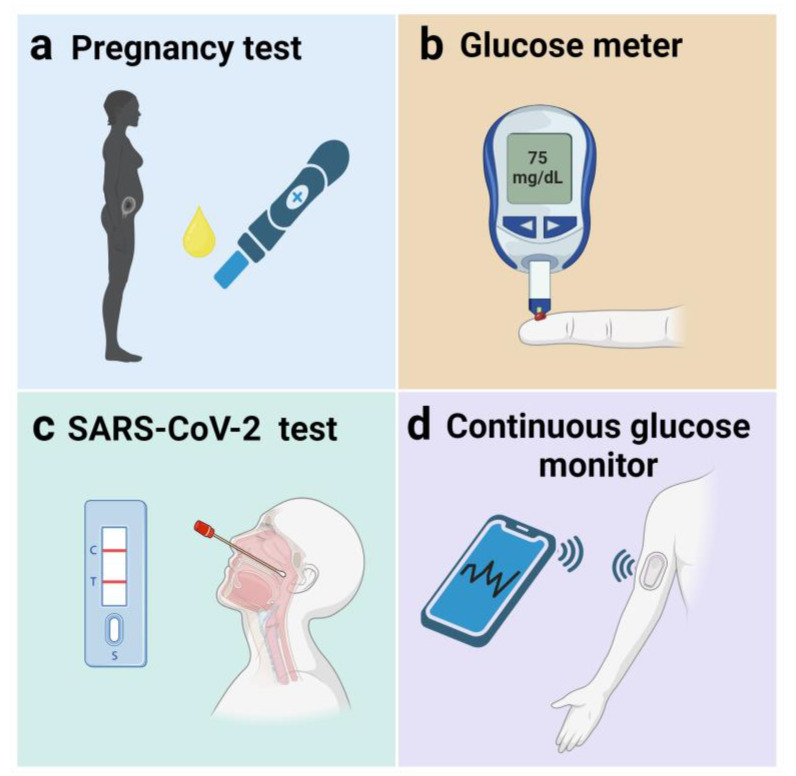
Commercial point-of-care biosensors for at-home use.

The concept of at-home POC sensing originated in the late 1970s with the commercialization of the first pregnancy tests [39]. To this day, pregnancy tests (Figure 2a) based on lateral flow assays of human gonadotropin (hCG) in urine remain one of the most widely available biosensors on the market [56]. Another widely commercialized biosensor is the personal glucose meter (Figure 2b), which has been on the market since the early 1980s [57]. These devices allow patients to monitor their blood glucose levels by themselves in a matter of seconds, enhancing diabetes management and reducing clinical visits. During the recent SARS-CoV-2 pandemic, the development of at-home viral detection kits (Figure 2c) played a crucial role in reducing the burden on the healthcare system and limiting transmission. These kits typically relied on lateral flow assays of SARS-CoV-2 surface proteins (e.g., spike, nucleocapsid proteins) [58]. In recent years, the development of body-based sensors (i.e., wearable and implantable devices) has exploded, offering enhanced at-home POC capabilities and continuous biomarker monitoring [2]. These devices rely on access to various biological fluids (e.g., interstitial fluid, sweat) to conduct on-body measurements and provide real-time information. Continuous glucose monitors (Figure 2d) have been particularly successful and comprise a significant fraction of the current biosensing market [16]. The deployment of POC biosensors for at-home use has transformed personal health management by ensuring medical monitoring is accessible and convenient. Not only does this improve management for chronic diseases like diabetes, but it helps to empower patients to proactively manage their own health in real and effective ways.

### 3.3. Sensing in the Field

The deployment of biosensors in the field represents another key area of POC assessment that can significantly enhance environmental and public health monitoring. Drug-of-abuse testing is one area that has benefited greatly from in-field biosensing strategies [59]. For example, rapid testing kits for dangerous substances like fentanyl can increase safety for first responders and assist medical and other personnel in identifying hazardous substances [60]. Another area of interest for in-field biosensors is the monitoring of water for harmful substances (e.g., heavy metals, bacterial pathogens). Biosensors have been proposed for the detection of these contaminants [61,62,63], which stand to greatly benefit impoverished communities that lack sufficient testing resources. Food safety monitoring can also be performed with biosensors, allowing for the identification of harmful pathogens (e.g., salmonella, *E. coli*) to reduce the risk of infection [15]. The deployment of infectious disease monitoring tools (e.g., SARS-CoV-2 biosensors) also enables the widespread tracking of disease incidence for public health purposes. Using this information, public health officials can enact various policies faster and better react to evolving public health situations [64].

### 3.4. Challenges in POC Sensing

While POC sensing technologies have greatly enhanced the accessibility and immediacy of medical diagnostics, they often cannot compare to the accuracy and reliability of traditional lab-based tests. This shortcoming primarily stems from inherent limitations in the design and functionality of POC devices. These devices typically use simplified methods and smaller sample sizes, often focusing on single-target detection, which may result in less accurate results. Additionally, the transducers in POC devices generally produce signals with lower sensitivity, partly because of the high levels of background noise, compared to the more sophisticated equipment used in laboratories. The use of POC devices in uncontrolled environments—such as home or field settings, where temperature, humidity, and user handling skills vary—introduces further variability that can compromise the test results. However, the future of POC technologies looks promising as advancements in AI continue to evolve. AI holds the potential to overcome these challenges by accelerating biomarker discovery and receptor development, enhancing data processing capabilities, improving device accuracy, and simplifying the operational complexities of POC tests. The integration of AI with biosensing technologies can dramatically improve the performance and reliability of POC diagnostics, bringing them ever closer to laboratory standards.

## 4. Artificial Intelligence: A Brief Overview

**Figure 3:**
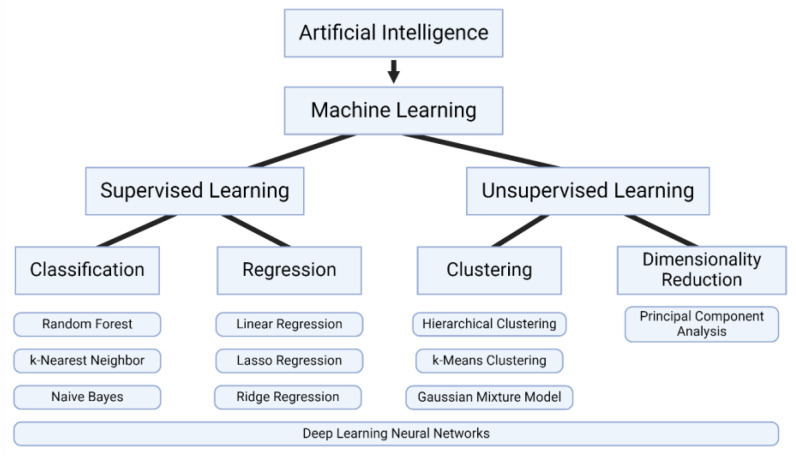
Overview of artificial intelligence approaches.

In the current AI landscape, machine learning stands as the main area of application in science (Figure 3) [65,66]. These methods involve the development of models through training algorithms with large sets of relevant data. Machine learning can be divided into two main areas depending on the nature of the data being used to train an algorithm. Supervised learning describes model training that uses characterized and labelled data. Unsupervised learning describes model training that uses uncharacterized data—where the model is left to draw inferences and recognize patterns on its own. In addition, reinforcement learning is a third area of machine learning that describes models that are trained on a trial-and-error basis by learning from their environment; however, given that reinforcement learning is more commonly applied to task-based scenarios (e.g., driverless cars), we will focus on supervised and unsupervised learning in this review.

Supervised learning can be further divided into classification or regression-based algorithms [67]. Classification algorithms serve to classify a given input dataset based on labels that were previously defined during training. Numerous classification algorithms have been proposed, all of which classify data using various determinative methodologies. The random forest algorithm generates randomized samples of a data pool (forests), with any given datapoint that can exist in multiple forests. The most common outcome of all samples (forests) is then selected. The k-nearest neighbor algorithm classifies a sample by assigning it to the category of the datapoint(s) nearest to it. The Naïve Bayes algorithm uses probability to classify a sample by treating all features as independent of one another and determining the most probable classification.

Regression-based algorithms serve to predict numerical values based on labelled data provided during model training. Linear regression is the simplest manifestation of this approach, which uses a fitted linear curve to estimate a dependent variable. More complex regression models include Ridge regression, Ordinal regression, and Lasso regression.

Unsupervised learning, in the context of biosensing, typically involves clustering or dimensionality reduction algorithms [68]. Clustering algorithms seek to group data based on similarities or learned characteristics. Hierarchical clustering groups samples such that samples in a group are more like one another than they are other groups. K-means clustering groups samples by assigning them to the cluster with the nearest mean. Gaussian mixture model clustering models each cluster as a Gaussian distribution and assigns samples to a cluster based on probability.

Dimensionality reduction-type algorithms focus on the simplification of data by identifying and maintaining the key features of a dataset. This allows a large dataset to be significantly reduced without losing important associations among samples. One such algorithm is principal component analysis, which applies a linear transformation to convert data into directional components that capture the most variation.

Deep Learning describes advanced machine learning algorithms that can operate independently, with little to no human intervention [69]. These methods are made possible by neural networks—systems of complex layers of neuron-like nodes capable of receiving, processing, and transmitting signals. Deep Learning approaches can be employed at any level of machine learning to provide higher functionality and more refined pattern recognition.

The use of algorithmic methods to train models can prove extremely powerful, but there are also many problems that can arise [70,71]. Overfitting occurs when a model is fit too closely to its training set, such that it cannot be generalized to new input data. Similarly, underfitting occurs when a model cannot capture the relationships and associations within a dataset. These scenarios, along with other issues relating to training data quality, group bias, and model scalability, can lead to spurious results without the user realizing. Thus, it is essential that users carefully select training data and validate models to reduce the risk of these complications.

## 5. Artificial Intelligence in Biosensing

When AI is discussed in the context of biosensing or other scientific fields, people tend to jump immediately to data analysis. While it is true that leveraging AI for data processing and analysis is powerful, that represents only a fraction of what AI is truly capable of. In fact, AI approaches can be applied at each stage of the biosensor development process, assisting in the selection of analytes, development of recognition elements, enhancement of signal transduction, and analysis and interpretation of data. Here, we describe many methods by which AI is being employed in the biosensing field, providing a clear picture of the power of these computational methods.

### 5.1. AI in Analyte Selection

At the analyte level, artificial intelligence is poised to enhance biosensing through the omics-driven elucidation of biomolecular pathways, the identification of novel biomarkers, and a combinatorial multi-analyte analysis. 

#### 5.1.1. Omics

The concept of omics is used to describe fields of study focused on the system-wide characterization of biological molecules (e.g., proteomics focuses on proteins, genomics on DNA, transcriptomics on RNA). Through the collective analysis of large quantities of these molecules, scientists aim to elucidate the mechanisms and processes that underpin human health and disease. Omics-driven analysis has proven especially useful in understanding complex diseases like cancer [72] and Alzheimer’s disease [73] that remain difficult to predict and treat. 

**Figure 4:**
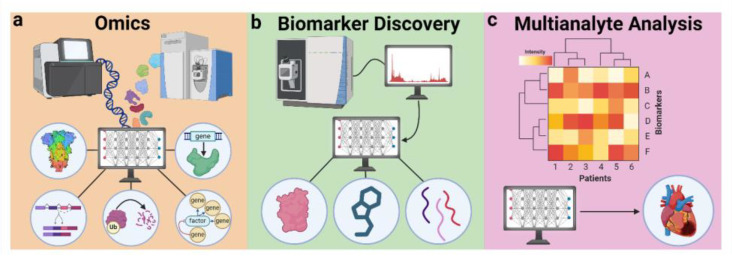
Artificial intelligence approaches for analyte discovery and understanding.

The data-heavy nature of omics makes it an excellent fit for integration with AI approaches, like machine learning [74], which help to uncover the structural and functional relationships among biomolecules (Figure 4a). AI-based tools have been developed to decipher gene regulatory pathways [75], estimate gene splicing [76], predict protein structure [77], predict protein degradability [78], and identify disease-driving genes [79,80], among many other applications. In addition, AI-omics approaches can be applied directly at the clinical level, for example, in the prediction of cancer metastasis [81] or to improve in vitro fertilization success [82]. AI also excels at clustering data into groupings that may not be obvious to the human eye—an ability that can prove vital in clinical settings. Using such classification-based approaches, AI-omics can accurately categorize diseases based on their omics profiles [83] and even predict individual treatment responses prior to drug administration [84].

The elucidation of biomolecular pathways with AI-integrated omics approaches provides a better understanding of human health and disease. In turn, this information allows for the design and deployment of biosensing technologies that are built-for-purpose to enable meaningful and practical biological measurements that directly improve patient care.

#### 5.1.2. Biomarker Discovery

The selection of relevant and practical biomarkers as analytes is a crucial part of the biosensor development process. The discovery of new biomarkers goes hand in hand with advances in omics, with a deeper understanding of molecular processes leading to more refined clinical targets. Using AI, researchers aim to identify biomarkers that strongly correlate with disease and are thus clinically and diagnostically useful. This is especially true for diseases like Alzheimer’s disease and dementia that currently lack robust biomolecular indicators [85]. 

The AI-assisted discovery of novel biomarkers can be accomplished by various machine learning avenues, including both classification and clustering (Figure 4b). Classification-type discovery involves finding biomarkers that can correctly identify a given disease state and has been accomplished using random forest [86], K-nearest neighbor [87], Naïve Bayes [88], and neural network [89] algorithms. Clustering-type discovery involves the grouping of patients based on biomarker profiles that can be analyzed using hierarchical clustering [90], K-means clustering [91], or Gaussian-mixture [92] algorithms. It is also common to combine multiple algorithms to improve biomarker quality [93] or to identify the most effective algorithm [88]. By applying AI techniques to high-throughput omics data, new biomarkers have been identified for a variety of diseases, including cancer [88], diabetes [94], and infectious diseases [95].

While improved biomarker identification methods can lead to better and more practical biosensors, the inverse also holds true. With the development of reliable, real-time biosensing capabilities—especially in wearable and implantable formats—it is possible to collect immense amounts of temporally resolved biomarker data [96]. The application of AI approaches to these real-time data can then allow for the determination of biomarker dynamics and relevance.

#### 5.1.3. Multianalyte Analysis

Most biosensors and clinical assays available today measure single biomarkers [97]. While single-target analysis can provide important information on human health (e.g., glucose sensors), the simultaneous measurement of multiple analytes allows for a more comprehensive assessment and enhanced clinical decision making [98]. With the discovery of new biomarkers comes a deeper understanding of the interconnectedness of the molecular world. While certain diseases may not have direct obvious biomarkers, it may prove possible to identify disease through a culmination of various biomolecular signatures. 

The assessment of multiple biomarkers is known as molecular profiling, which provides a bigger-picture view of individual health (Figure 4c). Molecular profiling is especially beneficial when individual biomarker fluctuations can be polycausal and difficult to diagnose (e.g., inflammatory responses [99]). Using profiling methods, it is possible to both diagnose disease [100] and predict individual treatment response [101,102] to various therapies. Biosensors for multiplexed profiling have been developed (e.g., for improved Alzheimer’s diagnosis [103]), but a lack of biochemical knowledge and issues with system integration have prevented the widespread adoption of these methods. Using AI, it is possible to recognize patterns in biochemical data to identify biomarker patterns that facilitate improved diagnosis at the POC [104,105,106]. For example, Lyme disease remains a difficult-to-diagnose disease due to its nonspecific or non-existent symptoms [107]. Through application of machine learning to identify optimal biomarkers, Joung et al. developed a paper-based biosensor capable of diagnosing Lyme disease in blinded samples with better success than previous POC tests [105].

### 5.2. AI in Recognition Element Selection

As diseases mutate and new health threats emerge, there is a pressing need for recognition elements that can sensitively and specifically identify a wide range of targets under diverse conditions. This demand has driven rapid progress in recognition element discovery, supported by several major methodological innovations, including the availability of rapid and low-cost DNA synthesis and deep sequencing services, the development of advanced bioinformatics tools, and the emergence of sophisticated experimental workflows for high-throughput function characterization. The incorporation of AI into recognition element identification has further revolutionized this field by enhancing screening methods for naturally occurring bioreceptors and even enabling the entirely de novo design of receptors in silico [108]. 

#### 5.2.1. Top-Down Discovery of Recognition Elements

**Figure 5:**
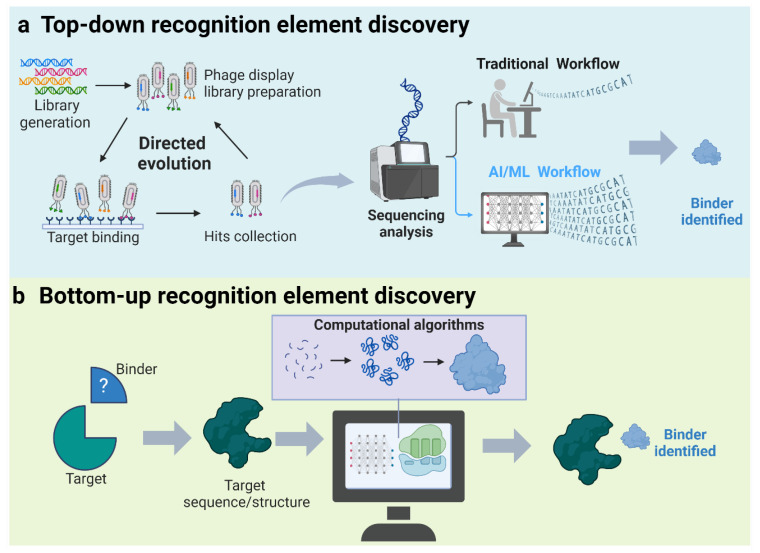
Artificial intelligence approaches to recognition element discovery.

High-throughput screening approaches, such as phage display, yeast display, and ribosome display for antibody discovery, along with SELEX for aptamer and nucleic enzyme discovery, have been widely used to identify new molecular-recognition elements for various targets [109,110,111]. However, these methods can be time-consuming, inefficient a costly, and often suffer from low success rates. AI has demonstrated great potential in addressing these challenges by efficiently processing vast amounts of screening data, ranging from thousands to millions of candidate interactions, to pinpoint the molecules most likely to exhibit the desired specificity and sensitivity (Figure 5a). Numerous AI-based tools have recently been developed to enhance biomolecular screening. For example, several tools have been developed to analyze high-throughput sequencing data from SELEX experiments in the form of cluster-based or motif-finding methods [112]. Cluster-based methods learn commonalities in aptamer candidates to group sequences into aptamer families; this allows for the identification of aptamers with common features (large families) that are likely stronger binders than aptamers that survived selection by chance (small families). Examples of cluster-based SELEX methods include AptaCluster [113] and FASTAptamer [114]. Motif-finding methods are designed to identify sequence or structural patterns indicative of target binding from a set of candidate aptamers. Examples of motif-finding methods include SMART-Aptamer [115] and CPS^2^ [116]. Beyond aptamer identification, machine learning algorithms, such as MLPL [117] and RaptGen [118], have been developed to optimize selected aptamers or even generate entirely new aptamers from sequencing data. At the same time, many machine learning tools for direct protein evolution [119], as well as antibody [120] and nanobody discovery [121], have been developed in recent years, significantly accelerating the generation of protein-based recognition elements. This progress can be found in recently published reviews [122,123,124]. 

#### 5.2.2. Bottom-Up Discovery of Recognition Elements

The emergence of AI approaches that allow for the accurate prediction of biomolecule structures directly from primary sequences, including nucleic acid or amino acid sequences, offers exciting new opportunities in the de novo design of recognition elements (Figure 5b). One of the most widely recognized tools is AlphaFold2 (AF2), an AI program developed by Google DeepMind, which has made over 200 million protein structure predictions to date [125,126]. This tool uses a deep neural network to assess the distances and angles between amino acid pairs within a protein sequence, using these geometrical constraints to accurately predict the three-dimensional structure of proteins. Building on the DeepMind framework, the Baker Lab developed RoseTTAFold (RF), a *three-track* neural network that simultaneously considers the patterns in protein sequences, interactions among protein amino acids, and potential three-dimensional protein structures, achieving structure predictions with accuracies comparable to those of DeepMind [127,128]. These tools enable provide a rapid solution to challenging X-ray crystallography and cryo–electron microscopy structure modelling problems and provide insights into the functions of proteins with currently unknown structures. 

The prediction of three-dimensional, single-stranded, nucleic acid structures, such as those of RNA, has also been explored with several recently developed tools, including Vfold [129], iFoldRNA v2 [130], and FARFAR2 [131]. The accurate modelling of protein–nucleic acid interactions has also recently become possible with the development of RoseTTAFold nucleic acid (RFNA) [132]. This single-trained network can rapidly produce three-dimensional structure models with confidence estimates for protein–DNA and protein–RNA complexes. This can be broadly useful for modelling the structure of naturally occurring protein–nucleic acid complexes, and for designing sequence-specific RNA and DNA-binding proteins. The recent release of RoseTTAFold All-Atom (RFAA) extends these capabilities, allowing for the modeling of assemblies that include proteins, nucleic acids, small molecules, and metals, as well as covalent modifications of these chemical structures [133]. Very recently, the AlphaFold 3 model was released with an updated diffusion-based architecture that is also capable of the joint structure prediction of complexes, including proteins, nucleic acids, small molecules, ions, and modified residues [134]. The Alpha Fold 3 model demonstrates significantly improved accuracy compared to many previous specialized tools: it achieves greater accuracy for protein–ligand interactions than state-of-the-art docking tools, higher accuracy for protein–nucleic acid interactions than nucleic acid-specific predictors, and higher antibody–antigen prediction accuracy than previous AlphaFold tools. 

Beyond the prediction of biomolecule structure, AI approaches have been employed to design proteins from scratch [135,136,137]. Such tools include ProteinMPNN [138], ProtGPT2 [139], and RoseTTAFold diffusion (RFdiffusion) [140]. By fine-tuning the RoseTTAFold structure prediction network, the Baker Lab developed a generative model of protein backbones that has an excellent performance for protein binder design, symmetric oligomer design, enzyme active site scaffolding, and symmetric motif scaffolding for therapeutic and metal-binding protein design [140]. The effectiveness and versatility of RFdiffusion has been validated through the experimental characterization of hundreds of designed symmetric assemblies, metal-binding proteins, and protein binders. High-affinity protein binders can be designed solely from the target sequence information [141], and are capable of binding diverse targets, including bioactive helical peptides [142], extended β-strand structures [143], amyloidogenic peptides [144], SARS-CoV-2 [145], and receptors for cytokines [108]. Additionally, the recent development of RFdiffusion All-Atom (RFdiffusionAA) enables the design of protein structures targeting small molecules [133]. Starting from random distributions of amino acid residues surrounding the target small molecules, this tool has successfully designed proteins that bind to therapeutics like digoxigenin, the enzymatic cofactor heme, and the light-harvesting molecule bilin, with a subset of these designs demonstrating the intended binding activity upon experimental validation.

AI approaches have also significantly advanced the de novo design of catalysts, including protein enzymes and nanozymes [146,147] that can function as recognition elements in biosensors. A notable example is the use of deep learning for the de novo design of artificial luciferases that are capable of oxidizing diphenylterazine with efficiencies that are comparable to native luciferases but with enhanced substrate specificity [148]. Advancements in AI-driven catalyst discovery have been summarized in several recently published reviews [149,150,151].

### 5.3. AI in Transduction

At the transduction level, artificial intelligence efforts focus on the intelligent design of new materials with enhanced biosensing properties, the miniaturization of existing sensing instrumentation, and the design of biosensing methods that transcend traditional biomolecular interactions.

#### 5.3.1. Material Design

**Figure 6:**
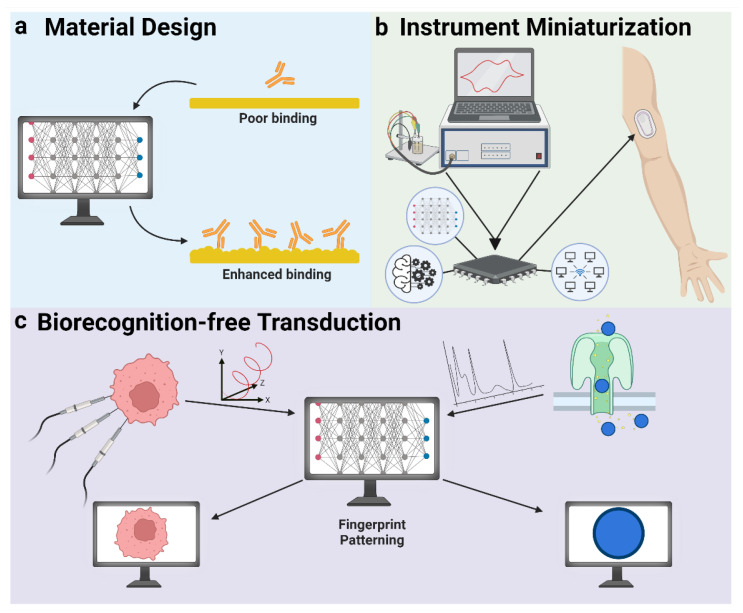
Artificial intelligence approaches to biosensor transduction.

The selection of compatible materials (e.g., electrode materials) is important in the development of highly sensitive and selective biosensors. Many modern optical systems rely on the incorporation of metamaterials—materials possessing various surface structures that allow for the control and modulation of electromagnetic radiation [152]. AI has been employed in the optimization and de novo design of new metamaterials to tailor optical properties for various applications (Figure 6a) [153,154]. For example, Malkiel et al. report a Deep Learning model capable of predicting plasmonic nanostructure geometries that produce optimal responses upon interaction with various biomarkers [155]. Similarly, machine learning models have been employed in the design of electrodeposited electrochemical sensors [156].

#### 5.3.2. Instrument Miniaturization

Biosensor transduction often requires the use of relatively bulky instrumentation; for example, spectrometers for optical measurements and potentiostats for electrochemical measurements. While efforts have been made to shrink these devices down to practical scales for POC or wearable applications, it remains difficult to sufficiently consolidate many instruments due to their complex components [157]. AI approaches to data collection and handling offer hope in this quest for miniaturization by providing enhanced computational abilities that can compensate for some loss in instrument complexity [158]. This allows sensing platforms to transduce signals with comparable resolution and efficacy to laboratory-based instruments (Figure 6b). For example, the emergence of computational spectrometers has allowed for the removal of traditional optical components (e.g., diffraction gratings) in favor of machine learning-driven algorithms capable of reconstructing spectral data with limited input [159,160].

#### 5.3.3. Biorecognition-Free Transduction

Recent years have seen increasing interest in the development of biorecognition-free biosensors that can detect biomolecules without the need for analyte-specific biological interactions [161]. The transduction of these sensors is often made feasible by employing machine learning algorithms capable of extracting patterns from seemingly non-specific data (Figure 6c). For example, Nicoliche et al. report a multidimensional sensor capable of profiling extracellular vesicle biomarkers based solely on capacitance measurements from five capacitors in parallel [162]. Using a machine learning model, they were able to extract a signal fingerprint that could detect the p16 protein down to 0.6 pg/mL. Nanopore-based biosensors represent another type of biorecognition-free sensor that can detect biomarkers through the electric current differences in molecules passing through nanoscale pores. Machine learning models can discriminate between various nanopore signals with high accuracy and have been used to detect a broad range of analytes [163,164,165].

### 5.4. AI in Data Handling

At the data-handling level, AI approaches focus on the generation of clean, detailed data that can provide robust insights for biomolecular analysis. Here, we analyze the role of AI at each stage of data management for POC biosensing and provide a brief overview of how AI technologies can be used to enhance biosensing during data acquisition, analysis, and interpretation.

#### 5.4.1. Data Acquisition

**Figure 7:**
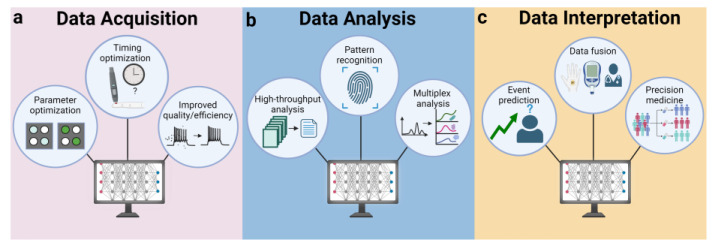
Artificial intelligence approaches for biosensor data handling.

During data acquisition (Figure 7a), sensor optimization algorithms can use reinforcement learning to adjust sensor parameters in real time and enable continuous calibration [166]. This optimization is important for biosensing systems in dynamic environments; for example, optical-based sensors may require the light intensity and exposure time to be adjusted to prevent sensor saturation and maximize the detection of fluorescent signals from biological markers. One such machine learning approach was developed to adjust optical parameters in real-time and enable a significantly enhanced performance and image quality [167]. The development of such feedback-driven systems provides a simple solution to variable environments without necessitating changes to sensor design.

AI can also improve the efficiency and quality of the data collected by biosensors. Biosensor data collection can prove cumbersome, especially when dealing with extremely large datasets and miniaturized devices with limited computing power. Dimensionality reduction algorithms offer promising solutions in these cases by providing an avenue to reduce data burden without compromising its diagnostic quality. For example, Ismaiel et al. reported one such algorithm that is capable of converting electrochemical impedance spectroscopy data—which are typically high-dimensional—into a one-dimensional sequence of values while maintaining the ability to distinguish changes in impedance behavior [168]. Another common challenge in biosensing is the existence of background noise that can mask the signal from the target analyte. Adaptive filtering techniques, such as those using neural networks, can distinguish between noise and the signal of interest with high accuracy [169,170]. These networks are trained to recognize the patterns associated with noise, allowing them to be filtered out without risking the elimination of target signal. 

AI-driven adaptive sampling techniques may also help to determine the optimal times and conditions under which samples should be collected to maximize information content and minimize resource use. This is particularly beneficial in POC applications, such as continuous glucose monitoring in diabetic patients, with AI algorithms being able to predict the best sampling times to extract medically relevant trends (e.g., sampling before and after lunchtime); this can reduce the frequency of unnecessary measurements while ensuring critical data are not missed.

#### 5.4.2. Data Analysis

At the data analysis stage (Figure 7b), machine learning algorithms can process and analyze vast amounts of data generated by biosensors, identify patterns, and classify data that might be missed by traditional methods. Machine learning algorithms such as random forests, neural networks, and decision trees can be trained on large datasets to rapidly classify and predict outcomes with high accuracy [171,172,173]. For example, Alafeef et al. report a neural network capable of predicting the cellular uptake of various nanoparticles by different cancers [174]. Using this model, along with eight different carbon nanoparticles, they were able to predict cancer type with an accuracy over 98%.

Machine learning algorithms have also been used in multiplex sensing assays, where multiple analytes are detected simultaneously. These methods can help to sort and analyze complex datasets to distinguish signals corresponding to different targets. For example, a neural network-powered optical biosensor was recently reported that can simultaneously detect multiple *Klebsiella pneumonia* bacterial biomarkers in real time with an accuracy of 99.8% [175].

#### 5.4.3. Data Interpretation

AI technologies also have the power to significantly enhance data interpretation (Figure 7c). They can handle the analysis of complex time-series data, such as data from continuous glucose monitoring or cardiac rhythm tracking, to predict future measurements or detect anomalous events based on historical data. This ability to analyze historical trends can help in the early detection of potential health issues, such as predicting hypoglycemic events in diabetic patients [176].

AI can also improve diagnostic accuracy by fusing data from heterogeneous biosensors [177]. For example, machine learning algorithms were employed to analyze mental fatigue levels with a predictive accuracy of up to 89% by evaluating six different kinds of physiological features using classification algorithms [178]. AI can also handle and analyze large volumes of diverse data—including electronic health records, medical images, wearable device outputs, genomics, and social media—which allows healthcare professionals to gain a holistic view of patient health [179,180]. This comprehensive data integration supports more precise diagnostics, tailored treatment plans, and proactive health management.

### 5.5. Artificial Intelligence Workflow

**Figure 8:**
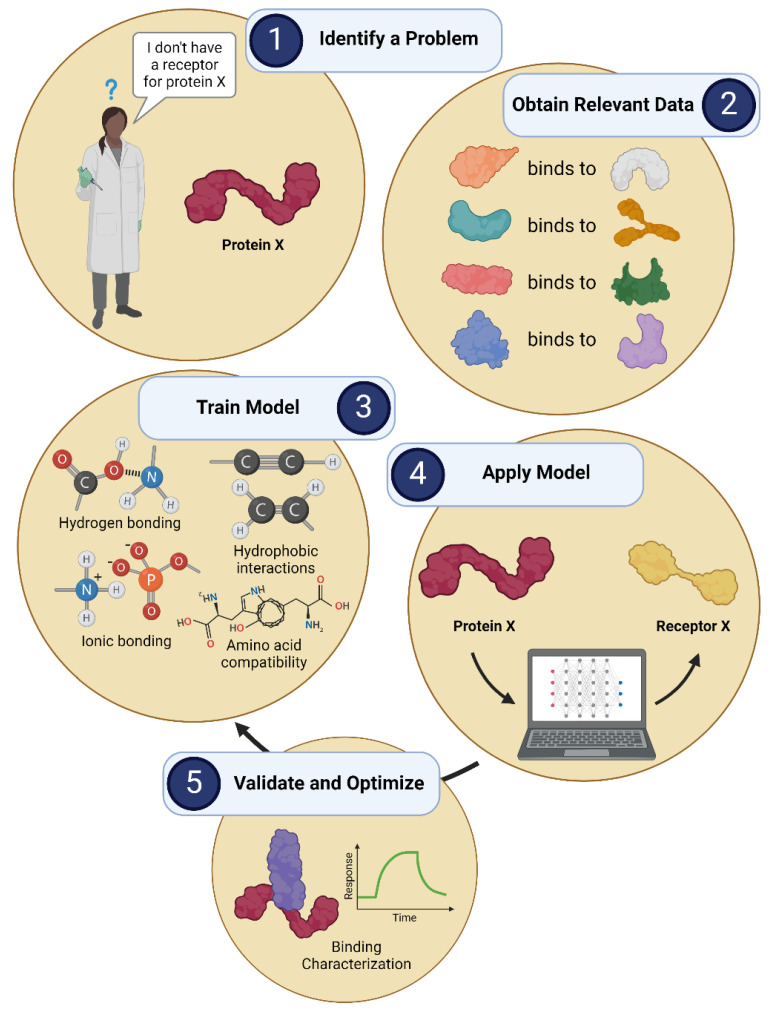
General workflow for artificial-intelligence-assisted model development.

AI is often purported to be a complex computational process that exists at an operating level far above what an average scientist is capable of learning. However, while the development and use of AI approaches may seem daunting at first glance, they are simply another scientific tool and should not invoke fear or aversion. In fact, all artificial intelligence approaches in science follow a similar workflow, which begins with identifying a problem and ends with solving that problem (hopefully). Understanding this workflow is key to understanding AI and integrating these approaches into your own research. This general workflow is described below. We have also included a schematic example in Figure 8 for the AI-assisted generation of recognition elements.

The first step in incorporating AI into your research is to *identify a problem*. These approaches are typically used to identify patterns or recognize features that the human mind is unable to easily determine; these tasks generally arise when there is an issue needing to be solved. It is important that the problem you identify is clear and concise, and that you have an actionable outcome in mind from the start. For example, a scientist may be developing a biosensor for a protein X that currently has no known biorecognition element.

The next step in the AI workflow is to *obtain relevant data*. While it may not be possible to obtain data directly related to your specific problem, you can find or collect data that can provide insight into the system you are looking to model. For example, the scientist may not have a receptor for protein X, but they do know of several other proteins that have known binding partners. Thus, they can collect information relating to these other protein-binding interactions for their model.

Once the data are in hand, it is time to *train the model*. To achieve this, researchers may use any number of available algorithms or develop their own from scratch. While some of these algorithms can be computationally complex, they all function in the same way: to identify patterns in the data that can be used to understand the intricate relationships between variables. For example, the model trained on the protein binding data may identify key molecular binding information (e.g., hydrogen bonding, amino acid compatibility) that will form the basis for predicting protein interactions.

Finally, it is time to *apply the model*. Here, the model trained on your dataset is put into action to test its effectiveness. The application of a model is almost never perfect on the first run, and it can take extensive effort to retrain and optimize the necessary parameters. The *validation and optimization* of your model should be carefully achieved with robust characterization and thoughtful data input. Once your model is capable of outputting legible data, you have succeeded in using AI to solve your problem. For example, the scientist will have successfully used AI once they can successfully apply their model to predict or design a receptor X for protein X. 

## 6. Challenges in AI POC Biosensing

The integration of AI into POC biosensing technologies offers new and exciting opportunities for expanded biosensing coverage and enhanced clinical decision making. Throughout this review, we highlighted many of the ways in which AI approaches are currently being implemented at each stage of the biosensor development process. This has allowed researchers to begin to address many of the significant issues facing current biosensors’ translation into clinical use. These issues include a lack of available high-quality biomarkers, a lack of available biorecognition elements, an inability to deploy certain instrumentation outside of laboratories, and an inability for sensors to adjust to dynamic environments. We summarize and report these challenges in Table 1. 

Like any emerging technology, the combination of AI and biosensing also faces many challenges that require careful consideration and robust solutions. For example, many biomolecule-oriented applications currently lack high-quality datasets that include standardized characterization methods. The added effects of inconsistencies in biosensor preparation, the limited shelf-life of biomolecules, and changes in the testing environment also contribute to widely variable data that are difficult to adequately compare. These factors can lead to issues like data overfitting or biases, causing models to fail to produce meaningful results. 

Moreover, many AI algorithms are designed with narrow application scopes, targeting specific diseases or conditions, which limits their universality and applicability across different medical domains. Most AI-biosensors are also not adaptive and do not learn from their environment after their initial programming. This limitation prevents them from adjusting to dynamic conditions (e.g., temperature changes) that can significantly diminish their effectiveness in real-world applications.

Cost and accessibility also pose significant challenges for AI integration. Many AI applications are computationally intensive and time-consuming, making them difficult to develop and implement outside of laboratory settings or by non-professional users. Access to high-quality datasets and computational resources can also be costly, which can restrict their use, especially in low-resource settings.

Furthermore, data privacy and security are critical as AI systems in POC devices handle sensitive health data, necessitating protection against unauthorized access and breaches. The opaque nature of many AI models also poses significant challenges in clinical settings, where trust in and understanding of diagnostic tools are crucial. The “black box” nature of these systems complicates their integration into medical practice as it is often difficult to explain how certain algorithms reach their conclusions. Regulatory challenges also arise, as the continual evolution of AI models requires a flexible regulatory approach that is not typically accommodated by traditional pathways, which complicates compliance and implementation efforts. Addressing these challenges is critical for the successful integration of AI into POC sensing and for realizing its full potential in improving healthcare outcomes. We summarize both the issues relating to implementing AI into biosensing and the issues with implementing AI approaches in general in Table 1 and Table 2, respectively.

## 7. Future Outlooks for AI-Assisted Biosensing

The combination of AI and biosensing technologies represents an important paradigm shift in the field. While many scientists are traditionalists who prefer methods they were trained in or methods they understand clearly, it is essential to recognize that AI approaches are here to stay. As years continue to pass, AI technology only continues to grow stronger and faster, and its incorporation into all avenues of scientific research is practically inevitable. It is never too late to learn new tools and we strongly encourage all readers to at least dip their toes into this exciting new area. The future of scientific research has never looked more promising than it does today, and AI-based methods will only continue to hasten this progress.

## Figures and Tables

**Table 1:** Challenges in AI-assisted POC biosensing.

		Biosensing Challenges				AI Biosensing Challenges		
Analyte								
		Lack of available biomarkers				Lack of high-quality training data		
		Lack of biomarker characterization data				Lack of open access omics data		
		Poor understanding of biomarker relationships				Limited disease-specific omics data		
		Many diseases without defined clinical tests						
Recognition Element								
		Lack of available receptors				Biased screening data		
		Lack of available catalysts				Lack of high-quality training data		
		Unknown biomolecular structures				Lack of standardized characterization		
		Inability to replicate natural receptors						
Transduction								
		Low material diversity for sensors				Hardware limitations		
		Need for biocompatible materials				Lack of sensor material data		
		Bulky instrumentation						
		Biomolecule instability & degradability						
Data Analysis								
		Excessive background noise				Lack of open access analysis programs		
		Inability to adjust to dynamic environments				Lack of generalizable models		
		Limited interpretative ability				Data privacy and security		
		Unable to consider outside factors						

**Table 2:** Challenges in AI approaches.

Development				
	Expensive and inaccessible datasets			
	Expensive computational resources			
	Biased data			
	Lack of standardized characterization methods			
Integration				
	Biased models			
	Overfitting or underfitting of data			
	Difficulty evaluating models for uncharted territories			
Deployment				
	Narrow application scopes; lack of universality			
	Abundance of repeated models			
	Issues with user trust			
	Lack of regulatory guidelines for evolving models			
	Lack of guidelines for user data			
	Lack of explainability			
